# Comorbidities and health‐care use prior to early‐onset Alzheimer's disease: A population‐based case–control study

**DOI:** 10.1002/alz.71831

**Published:** 2026-10-08

**Authors:** Laura Leppänen, Adolfina Lehtonen, Mikko Aaltonen, Kalle Aho, Ave Kivisild, Abbe Ullgren, Iina Rinnankoski, Helmi Soppela, Annakaisa Haapasalo, Anne M. Portaankorva, Päivi Hartikainen, Kasper Katisko, Eino Solje, Johanna Krüger

**Affiliations:** ^1^ Research Unit of Clinical Medicine, Neurology University of Oulu Oulu Finland; ^2^ Law School University of Eastern Finland Joensuu Finland; ^3^ Institute of Clinical Medicine–Neurology University of Eastern Finland Kuopio Finland; ^4^ Department of Neurobiology, Care Sciences and Society, Division of Neurogeriatrics, Karolinska Institutet Solna Sweden; ^5^ A.I. Virtanen Institute for Molecular Sciences, University of Eastern Finland Kuopio Finland; ^6^ Clinical Neurosciences University of Helsinki Helsinki Finland; ^7^ Neuro Center–Neurology Kuopio University Hospital Kuopio Finland; ^8^ Neurocenter, Neurology Oulu University Hospital Oulu Finland; ^9^ Medical Research Center Oulu University Hospital Oulu Finland

**Keywords:** Alzheimer's disease, brain injury, comorbidity, early‐onset dementia, epilepsy, health‐care visit, otorhinolaryngologic disease, thyroid disorder

## Abstract

**INTRODUCTION:**

We examined whether comorbidities and health‐care use differ between individuals later diagnosed with early‐onset Alzheimer's disease (EOAD) and matched controls during the 15 years preceding diagnosis.

**METHODS:**

This population‐based case–control study included 407 EOAD patients diagnosed at two Finnish university hospitals (2010–2021), and 4035 age‐ and sex‐matched controls. Prior diagnoses and health‐care visits were obtained from nationwide registers.

**RESULTS:**

EOAD patients had higher prevalence of vertigo (5.9% vs. 2.1%, *p* < 0.001), depression (5.7% vs. 2.3%, *p* < 0.001), headache (4.9% vs. 1.6%, *p* < 0.001), delirium (1.5% vs. 0.1%, *p* < 0.001), brain injuries (2.9% vs. 0.9%, *p* = 0.002), thyroid disorders (2.5% vs. 0.8%, *p* = 0.008), alcohol‐related disorders (3.4% vs. 1.4%, *p* = 0.009), epilepsy (2.2% vs. 0.7%, *p* = 0.009), strokes (4.4% vs. 2.0%, *p* = 0.008), and otorhinolaryngologic diseases (5.2% vs. 2.9%, *p* = 0.027) 1–5 years before diagnosis. Only otorhinolaryngologic diseases (4.7% vs. 2.1%, *p* = 0.036) differed 6 to 10 years prior, whereas no differences were seen 11 to 15 years before diagnosis. Hypertension and dyslipidemia showed no between‐group differences. Health‐care use increased markedly from year before EOAD diagnosis.

**DISCUSSION:**

EOAD is preceded by recognizable patterns of morbidity and increased health‐care use, which may support earlier detection.

## Highlights

- Early‐onset Alzheimer's disease (EOAD) is preceded by a distinct profile of comorbidities.
- Depression, delirium, brain injuries, and epilepsy increased 1 to 5 years pre‐diagnosis.
- Traditional cardiovascular risk factors were not associated with EOAD.
- Health‐care service use increased markedly during the year preceding diagnosis.
- Antecedent morbidity patterns may aid earlier recognition of EOAD.

## 1 BACKGROUND

Alzheimer's disease (AD), the most common cause of neurodegenerative dementia, accounts for 60% to 70% of all dementia cases.^1^ Major risk factors include age, female sex, and the presence of at least one apolipoprotein E ε4 allele.^2^, ^3^ Cardiovascular risk factors and unhealthy lifestyle behaviors have been associated with an increased risk of late‐onset AD (LOAD), while lower education attainment significantly correlates with heightened susceptibility to both early‐onset AD (EOAD) and LOAD.^4^, ^5^

EOAD, defined by symptom onset before the age of 65, accounts for up to 10% of all AD cases.^1^ EOAD differs from LOAD in several ways, including a higher prevalence of atypical clinical presentations and a more aggressive disease trajectory.^6^ Patients with EOAD typically experience shorter survival times and longer diagnostic delays, with an average time to diagnosis of 41.8 months compared to 30.6 months in LOAD.^7^, ^8^ Furthermore, EOAD is suggested to associate with a lower burden of comorbid conditions compared to LOAD.^9^ However, the comorbidity profile of EOAD remains insufficiently characterized, with only a limited number of studies addressing this aspect.^9^, ^10^

This study investigated the prevalence of comorbid medical conditions and diagnoses in EOAD patients during three pre‐diagnostic intervals: 1 to 5, 6 to 10, and 11 to 15 years before dementia diagnosis. Comparisons were made to age‐ and sex‐matched controls without a diagnosis of EOAD from the same geographic regions. Longitudinal trends in use of health‐care services were also assessed by quantifying and comparing the number of health‐care service visits between EOAD patients and controls over the 15‐year period preceding the diagnosis.

## 2 METHODS

This study was approved (THL/2841/14.02.00/2022) by the Finnish Social and Health Data Permit Authority Findata (www.findata.fi/en). According to the Finnish legislation (552/2019), ethics board review or patient consent were not required because only registry data were involved in the study without contacting the patients. This study is part of DEGE‐RWD research project (protocol registered to ClinicalTrials.gov: NCT06209515), coordinated by Neurocenter Finland.

All patients with a diagnosed or suspected progressive neurodegenerative disease referred to the memory clinics of Oulu University Hospital (OUH) and Kuopio University Hospital (KUH) between 2010 and 2021 were identified from the university hospitals’ patient registries. These registries are linked to the electronic health record systems, using International Classification of Diseases 10th Revision (ICD‐10) codes. An ICD‐10 code search covering 156 codes initially yielded 12,490 cases. In Finland, all suspected early‐onset neurodegenerative cases, regardless of socioeconomic status, are systematically referred to the regional university hospital (OUH for Northern Ostrobothnia, KUH for Northern Savonia), ensuring population‐based coverage. Clinical data from medical records for all cases were reviewed to ensure that the cases fulfilled the standardized AD criteria.^11^ After re‐evaluation, 407 patients with EOAD (diagnosis ≤ 65 years) were included. Diagnostic procedures included structural magnetic resonance imaging (MRI) and comprehensive neuropsychological assessment for all cases, cerebrospinal fluid (CSF) AD biomarker analysis, or amyloid positron emission tomography (PET) when clinically indicated. Of the patients with EOAD in this study, 62.2% had CSF amyloid and tau biomarkers measured, indicating AD pathology.^12^ Amyloid PET was performed on only a few cases of this study.

**RESEARCH IN CONTEXT**

1. **Systematic review**: Previous early‐onset Alzheimer's disease (EOAD) studies have examined cardiovascular, metabolic, psychiatric, endocrine, and infectious risk factors, yet few studies have evaluated long‐term comorbidity patterns and health‐care use preceding diagnosis. Most dementia research focuses on late‐onset Alzheimer's disease (LOAD), linking it to hypertension, dyslipidemia, and metabolic syndrome. In contrast, evidence regarding EOAD remains comparatively limited and heterogenous.
2. **Interpretation**: Our findings show that EOAD is associated with higher prevalence of vertigo, depression, headache, delirium, brain injuries, thyroid disorders, alcohol‐related disorders, epilepsy, strokes, and otorhinolaryngologic diseases, especially 1 to 5 years before diagnosis. In contrast, common cardiovascular conditions were not increased. EOAD patients also used health‐care services more frequently near diagnosis, suggesting prodromal features and potential diagnostic delays. Overall, EOAD displays a distinct comorbidity pattern compared to LOAD and may be less influenced by vascular risk factors.
3. **Future directions**: Longitudinal studies are needed to clarify causal links between comorbidities and EOAD. Identifying prodromal comorbidity patterns could improve early detection and guide targeted interventions.

After case validation and cohort formation, the study cohort was linked to nationwide health registers using personal identification numbers to obtain data on comorbidities and health‐care use. Comorbidities were defined based on clinically recorded ICD‐10–coded diagnoses in national health registers according to standard clinical diagnostic practice. ICD‐10–coded diagnoses were grouped into predefined phenotypic categories, and all diagnoses observed within these categories among EOAD patients during the pre‐diagnostic period were included in the analyses. To comply with Finnish data privacy legislation, we first inspected all ICD‐10 codes that appeared in the EOAD group for at least five individuals during the entire pre‐diagnostic period. These were further merged to 23 predefined diagnostic categories that were analyzed across three pre‐diagnostic time periods (1–5, 6–10, and 11–15 years before EOAD diagnosis). The comorbidities of EOAD patients were categorized according to their respective domains. The definitions of the diagnostic categories were based on ICD‐10 coding practices used in clinical health care. In Finnish health care, it is mandatory to include relevant ICD codes to medical records after each health‐care visit. For example, diabetes was defined based on a recorded clinical diagnosis made according to standard criteria also defined in the Finnish Current Care Guidelines for diabetes^13^, including fasting plasma glucose ≥ 7.0 mmol/L, 2‐hour plasma glucose > 11.1 mmol/L during an oral glucose tolerance test, or HbA1c ≥ 48 mmol/mol (6.5%) in asymptomatic individuals, as well as a random plasma glucose > 11.0 mmol/L in symptomatic patients. Depression was based on ICD‐10 clinical diagnostic criteria,^14^ requiring a minimum duration of depression symptoms of at least 2 weeks, with clinically significant impairment in functioning.

The thyroid disorder group included hypothyroidism and thyrotoxicosis associated with diffuse goiter. The gastrointestinal disease group included gastroesophageal reflux disease, esophagitis, and diverticular disease of the colon without bowel perforation or abscess. The group of otorhinolaryngologic diseases included tinnitus and bilateral sensorineural hearing loss. The musculoskeletal disorder group included spinal stenosis, intervertebral disc disease with radiculopathy, disc degeneration, cervical brachial syndrome, rotator cuff syndrome, and shoulder impingement syndrome. The infection group included infectious diarrhea and gastroenteritis illnesses, erysipelas, pneumonia, and postoperative infections. The group of heart disorders included atherosclerotic heart disease, mitral valve prolapse, atrial fibrillation or atrial flutter, persistent atrial fibrillation, and unspecified heart arrhythmia. The stroke group included cerebral infarction, late effects of cerebral infarction, and transient ischemic attack. The other injuries group included fracture of the upper end of the humerus, injury of a rotator cuff tendon, fracture of the lower end of the radius, fracture of the lateral malleolus, and acute meniscal tear of the knee. The group of cardiovascular risk factors included hypertension and dyslipidemia.

In Finland, documentation of each health‐care service encounter, whether in public or private settings, requires the recording of a primary diagnosis using ICD‐10 codes. These diagnostic codes, along with any secondary diagnoses, have been systematically transmitted to the national Care Register for Health Care since 1994 maintained by the Finnish Institute for Health and Welfare. The register encompasses data from inpatient care, day surgery procedures, and outpatient visits in public specialized medical services. Consequently, all diagnoses ever done for both the EOAD patients and matched controls across these care settings were available for inclusion in this study.

The control group included 4035 age‐ and sex‐matched individuals without a progressive neurodegenerative disease from the same regions, randomly sampled by the Digital and Population Data Services Agency. Controls were drawn using cumulative density sampling, so cases could not be selected before diagnosis. We compared the prior medical diagnoses of the EOAD patients at three intervals—1 to 5, 6 to 10, and 11 to 15 years before diagnosis—to matched controls. Diagnosis‐specific binary variables indicated the presence or absence of each diagnosis. An annual variable was also generated for the number of distinct care episodes recorded in the health‐care register for both groups.

### 2.1 Statistical analyses

The statistical significance of differences between patients and controls in the binary variables indicating different medical diagnoses were examined using conditional logistic regression models for each diagnosis type separately, with group indicator (control = 0, patient = 1) as the outcome. These models allow for variable matching ratio and restrict the comparison of each patient to their controls only. The model results are reported as odds ratios and their 95% confidence intervals. To control for multiple testing, we adjusted the *p* values from regression models using Benjamini–Hochberg false discovery rates (FDRs) using the R package “FDRestimation.”^15^, ^16^ Adjustment was done separately for three groups of estimates by time interval. We report the FDR‐adjusted *p* values together with time‐interval–specific proportions of patients and controls with different comorbidities. Due to data privacy rules of Statistics Finland, we only report differences for diagnoses when the cell count in each group is ≥ 3. The statistical significance of differences in mean number of care episodes was examined using a *t* test.

## 3 RESULTS

Our study included 407 patients diagnosed with EOAD and 4035 matched controls. The characteristics of the study population are presented in Table 1. The groups were matched by age and sex; in both groups 41.5% of the subjects were male and 58.5% female. The mean age at the time of EOAD diagnosis was 60.6 years (standard deviation = 4.5). There were no significant differences in the education level in the groups. Of the subjects, 35.6% were from KUH and 64.4% from OUH district.

**TABLE 1:** Basic characteristics of the study cohort and controls.

	EOAD (*n* = 407)	Controls (*n* = 4035)
Year of diagnosis		
Mean (SD)	2015.7 (3.9)	
Median	2016	
Age at diagnosis, years		
Mean (SD)	60.6 (4.5)	60.6 (4.5)
Median	62	62
Sex		
Male %	41.5	41.5
Female %	58.5	58.5
Area		
KUH %	35.6	35.8
OUH %	64.4	64.2

Abbreviations: EOAD, early‐onset Alzheimer's disease; KUH, Kuopio University Hospital; OUH, Oulu University Hospital; SD, standard deviation.

All the diagnoses set for both subgroups at 1 to 5 years, 6 to 10 years, and 11 to 15 years before the EOAD diagnosis are reported in Table 2. The odds ratios of various comorbidities across the pre‐diagnostic intervals are presented in Figure 1. Across all examined time points, EOAD patients showed a progressive increase in the number of diagnosed disorders, particularly 1 to 5 years before diagnosis, in contrast to controls. Within the 1 to 5 years preceding EOAD diagnosis, several comorbid conditions were significantly more prevalent in EOAD patients compared to controls. The most statistically significant differences were observed for vertigo (5.9% in EOAD vs. 2.1% in controls, *p* < 0.001), depression (5.7% vs. 2.3%, *p* < 0.001), headache (4.9% vs. 1.6%, *p* < 0.001), and delirium (1.5% vs. 0.1%, *p* < 0.001). Additional significant differences were observed for brain injuries (2.9% vs. 0.9%, *p* = 0.002), thyroid disorders (2.5% vs. 0.8%, *p*  = 0.008), strokes (4.4% vs. 2.0%, *p* = 0.008), alcohol‐related disorders (3.4% vs. 1.4%, *p* = 0.009), epilepsy (2.2% vs. 0.7%, *p* = 0.009), and otorhinolaryngologic diseases (5.2% vs. 2.9%, *p* = 0.027).

**TABLE 2:** Prevalence of other diseases and symptoms in EOAD patients and controls prior to the EOAD diagnosis.

	1–5 years before, %			6–10 years before, %			11–15 years before, %		
	EOAD	Controls	*p* value	EOAD	Controls	*p* value	EOAD	Controls	*p* value
Asthma	1.5	2.6	0.326	2.5	1.6	0.583	2.5	1.5	0.375
Thyroid disorders	2.5	0.8	0.008^*^						
Depression	5.7	2.3	<0.001^*^	2.7	2.5	0.885	2.2	2.4	0.887
Arthrosis	6.4	6.8	0.806	5.2	5.3	0.939	1.2	2.8	0.236
Gastrointestinal disorders	3.7	2.7	0.425	2.7	2.0	0.583	1.7	1.5	0.887
Unspecified dermatitis	1.5	0.9	0.466	1.0	0.9	0.885			
Otorhinolaryngologic diseases	5.2	2.9	0.027^*^	4.7	2.1	0.036^*^	1.7	1.3	0.887
Senile cataract	1.2	1.8	0.543	0.7	0.6	0.885			
Musculoskeletal disorders	9.3	9.1	0.908	10.3	9.9	0.885	6.4	7.1	0.887
Alcohol‐related disorders	3.4	1.4	0.009^*^	1.5	1.1	0.885			
Infections	7.6	5.6	0.228	6.4	4.4	0.430	2.9	2.5	0.887
Heart disorders	7.6	6.0	0.354	4.2	3.2	0.583	2.5	2.1	0.887
Diabetes (type I and II)	3.2	2.6	0.605	3.2	1.9	0.430	2.2	1.0	0.103
Cardiovascular risk factors (hypertension or dyslipidemia)	10.6	9.3	0.543	4.7	4.8	0.918	2.0	1.8	0.887
Sleep apnea	3.4	4.2	0.571	1.2	2.5	0.576	1.5	1.4	0.887
Epilepsy	2.2	0.7	0.009^*^	1.5	0.6	0.430	1.2	0.3	0.103
Strokes	4.4	2.0	0.008^*^	1.5	1.2	0.885			
Headache	4.9	1.6	<0.001^*^	2.5	1.7	0.583	1.7	2.0	0.887
Vertigo	5.9	2.1	<0.001^*^	2.2	2.4	0.885	2.9	1.4	0.103
Delirium	1.5	0.1	<0.001^*^						
Pain	10.1	9.3	0.700	9.6	8.8	0.885	7.6	8.0	0.887
Brain injuries	2.9	0.9	0.002^*^	1.2	0.7	0.583			
Other injuries	4.2	3.4	0.543	4.7	3.4	0.583	2.7	2.7	0.981
*N*	407	4035		407	4035		407	4035	

*Note*: False discovery rate–corrected *p* values, initial estimates from conditional logistic regression models.

Abbreviation: EOAD, early‐onset Alzheimer's disease.

^*^Statistically significant association at 95% confidence level.

**FIGURE 1:**
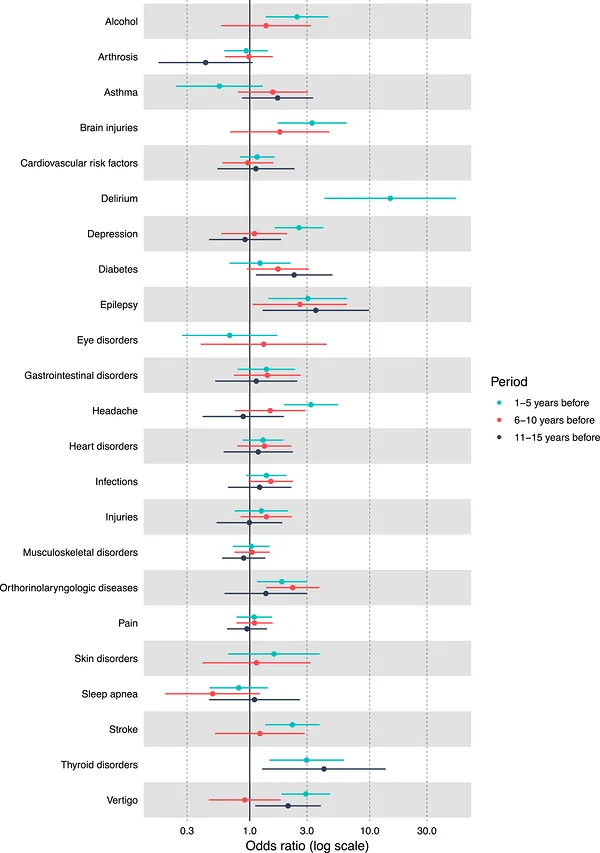
Odds ratios for comorbidities among EOAD patients compared to matched controls across three pre‐diagnostic time intervals (1–5, 6–10, and 11–15 years before EOAD diagnosis). EOAD, early‐onset Alzheimer's disease.

During the 6 to 10 years preceding EOAD diagnosis, otorhinolaryngologic diseases were significantly more common in the EOAD group than in controls (4.7% vs. 2.1%, *p* = 0.036). No other significant group differences were observed during this period. During the 11 to 15 years preceding diagnosis, none of the examined comorbidities differed significantly between EOAD patients and controls.

We then compared the frequency of health‐care service visits between the EOAD patients and matched controls during the years preceding the diagnosis. In line with the observed increase in diagnostic burden near the time of EOAD diagnosis, EOAD patients exhibited a significantly higher number of health‐care service visits (Figure 2) during the year immediately preceding the diagnosis (mean 3.3 vs. 1.9, *p* < 0.001) and in the year of the diagnosis (mean 6.3 vs. 2.0, *p* < 0.001). No statistically significant differences were observed ≥ 2 years prior to diagnosis (Figure 2).

**FIGURE 2:**
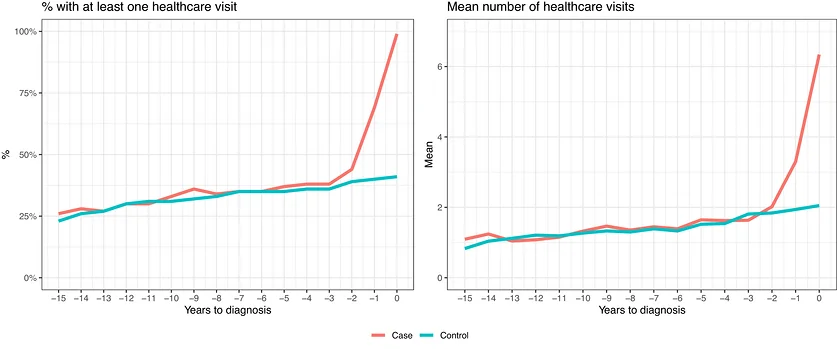
Proportion of individuals with at least one health‐care visit and mean number of visits among EOAD patients and matched controls prior to EOAD diagnosis. EOAD, early‐onset Alzheimer's disease.

## 4 DISCUSSION

This study examined diagnosed medical conditions during the 1 to 5, 6 to 10, and 11 to 15 years preceding EOAD diagnosis compared to matched controls. The most pronounced differences were observed during the 1 to 5 years before diagnosis, when EOAD was significantly associated with increased prevalence of vertigo, depression, headache, delirium, brain injuries, thyroid disorders, strokes, alcohol‐related disorders, epilepsy, and otorhinolaryngologic diseases. During the 6 to 10–year period, only otorhinolaryngologic diseases differed between groups, whereas no significant differences were observed 11 to 15 years before diagnosis. Although partially consistent with previous findings on AD risk factors, increased cardiovascular or metabolic burden was not detected in EOAD patients. The number of comorbid diagnoses and health‐care visits increased closer to EOAD diagnosis, particularly during the year before and at the year of the diagnosis. These findings may reflect diagnostic delays and challenges in symptom recognition, but may also result from early symptom progression, differences in care‐seeking behavior, or health‐care system–related factors.

Interestingly, established cardiovascular risk factors, such as hypertension and dyslipidemia, were not more prevalent in EOAD patients than in matched controls, opposite to the findings in LOAD.^17^, ^18^ Hypertension has been associated with an increased risk of LOAD through mechanisms including vascular remodeling, hypoperfusion, ischemia, and hypoxia.^19^ Despite previous reports linking elevated levels of low‐density lipoprotein cholesterol (LDL‐C) with an increased EOAD risk,^20^, ^21^, ^22^ we found no association between EOAD and established vascular risk factors. Only one previous smaller study has reported similar findings. Direct assessment of cardiorespiratory fitness was not possible in our registry‐based study. Although low or moderate fitness at the age of 18 has been associated with an increased risk of early‐onset dementia,^23^ our findings did not support a higher prevalence of established vascular risk factors among EOAD patients. This may suggest that EOAD differs from LOAD in its underlying etiological profile.^20^

Only a few studies have examined prior medical conditions or health‐care service use before the EOAD diagnosis. EOAD has been linked to circulatory, psychiatric, behavioral, endocrine, and metabolic disorders—particularly hypertension, metabolic syndrome, and diabetes—with prevalences of 4.5% to 20.3%.^9^ Infections requiring treatment at a hospital have also been associated with EOAD.^24^ In our cohort, infections such as diarrheal and gastroenteritis illnesses, erysipelas, pneumonia, and postoperative infections appeared more frequent in EOAD patients than in controls, even though the associations were not statistically significant. Registry data did not allow assessment of infection severity. Nevertheless, the increased frequency of infections may reflect underlying immune‐related processes in the prodromal phase of the disease. However, potential confounding factors, such as differences in health‐care use and diagnostic intensity, may also have influenced these findings, and further studies are needed to clarify the association.

Our results are consistent with previous studies reporting an association between traumatic brain injury (TBI)—including concussion and subdural hematoma—and EOAD, and severe TBI with increased risk of early‐onset dementia.^25^, ^26^ Our findings also align with the evidence stating that strokes are associated with an increased risk of AD.^27^ Moreover, adults aged 45 to 64 with traumatic spinal cord injury show a 2‐fold higher EOAD risk.^28^ Altogether, these findings suggest that neurological trauma may be involved in EOAD pathophysiology, potentially through mechanisms including neuroinflammation, vascular disruption, and impaired neural repair.^29^, ^30^

In our study, epilepsy was frequently observed in EOAD patients 1 to 5 years before diagnosis. This is consistent with previous research reporting epilepsy in up to 40% of EOAD cases and associating comorbid epilepsy to more severe disease progression and accelerated cognitive decline.^31^ Seizures have also been associated with AD, especially familial EOAD.^32^, ^33^ Proposed mechanisms include amyloid beta (Aβ) pathology, generalized epilepsy, hippocampal sclerosis, and shared tau‐related processes.^34^, ^35^, ^36^, ^37^ While causality remains uncertain, epilepsy may represent both an early clinical marker and a comorbid condition associated with EOAD, highlighting the importance of cognitive monitoring in individuals with epilepsy.

Delirium was frequently observed during the 1 to 5 years preceding EOAD diagnosis, consistent with prior findings reporting an increased risk of delirium in individuals with dementia.^38^ Recent Mendelian randomization analyses suggest that delirium does not causally increase the risk of AD. Rather, underlying AD pathology may predispose individuals to delirium.^39^ These findings support the interpretation that delirium may reflect early neurodegenerative changes and may serve as a clinical indicator of prodromal EOAD.

Depression, in addition to delirium, was more prevalent in EOAD patients 1 to 5 years before diagnosis compared to controls. Depression has been suggested as both a potential risk factor and a prodromal symptom of dementia.^40^, ^41^, ^42^ Anxiety and depression are common in EOAD, possibly related to rapid disruption of daily functioning.^43^, ^44^ These findings highlight the importance of monitoring mood symptoms as potential early indicators of cognitive decline.^41^, ^42^ However, these associations are observational and may reflect prodromal disease rather than a causal association.^41^, ^42^

In our cohort, alcohol‐related disorders, including alcohol dependence syndrome and delirium tremens without seizures, were significantly more prevalent during the 1 to 5 years preceding EOAD diagnosis. Previous research has suggested that heavy alcohol consumption may be associated with accelerated cognitive decline in AD.^45^ Increased alcohol use prior to diagnosis may reflect prodromal neurodegenerative or neuropsychiatric changes, although it may also represent an independent risk factor.

Thyroid disorders, including hypothyroidism and thyrotoxicosis with diffuse struma, were significantly more common in EOAD patients 1 to 5 years before diagnosis. Previous studies on thyroid function and AD onset have been inconclusive due to small cohort sizes and methodological limitations, although low thyroid‐stimulating hormone and high free T4 levels have been linked to increased dementia risk.^46^, ^47^ Our findings suggest a potential association between thyroid disorder and EOAD.

Tinnitus and bilateral sensorineural hearing loss were more common in EOAD patients 1 to 10 years before diagnosis. These findings align with previous studies reporting an association between tinnitus and increased AD risk.^48^ Midlife hearing loss has also been identified as being associated with an increased risk of dementia, possibly due to reduced cognitive reserve and greater cognitive load in demanding auditory settings.^49^ Aβ and tau accumulation, together with related cortical pathology and atrophy, may further be associated with impaired auditory processing and cognitive decline.^50^, ^51^, ^52^, ^53^

We also observed the EOAD patients had significantly more health‐care service visits during the year of diagnosis and the preceding year than matched controls, while no differences were detected earlier. This increase may reflect early, non‐specific symptoms, such as headache, vertigo, or depression, which may be related to subtle neurological changes or may be influenced by autonomic dysfunction or somatization.

To our knowledge, this is the largest study to date examining prior medical conditions 1 to 15 years before EOAD diagnosis, using a population‐based cohort and age‐ and sex‐matched controls from the same geographic region. All EOAD cases diagnosed at two university hospital memory clinics by experienced neurologists over a 12‐year period were included, and diagnoses were retrospectively re‐evaluated to enhance specificity. These hospitals serve as regional referral centers for suspected early‐onset dementia cases, ensuring comprehensive case ascertainment independent of socioeconomic status. A major strength of this study is Finland's integrated health‐care system, which enabled collection of diagnostic data from public and private health‐care providers. Matched controls without neurodegenerative diseases were drawn from the same population base, minimizing selection bias. The longitudinal study design, with multiple pre‐diagnostic time points, strengthens internal validity and enables temporal assessment of disease patterns and health‐care use.

This study also has some limitations. First, we did not account for competing risks, as only individuals surviving through each pre‐diagnostic interval were included, potentially introducing survivor bias. Second, the observational and exploratory design limits causal inference, and residual confounding cannot be excluded. EOAD was defined based on age at diagnosis rather than symptom onset, which is difficult to determine retrospectively and may have excluded individuals with onset close to age 65 years but diagnosis after this age. Socioeconomic, lifestyle, and genetic factors were unavailable in registry data, and although *p* values were adjusted for multiple testing, findings should be validated in other datasets. Third, comorbid diagnoses from national health‐care registers could not be independently verified, and some misclassification or incomplete recording is possible. However, mandatory diagnostic coding and comprehensive health‐care coverage support the reliability of population‐level estimates. Register‐based data did not allow assessment of disease severity, functional status, or care intensity. CSF biomarkers were available for ≈ 62% of cases, reflecting real‐world clinical practice during 2010 to 2021. Because controls were classified based on the absence of diagnosed neurodegenerative disease during follow‐up, undetected preclinical pathology cannot be excluded. Finally, the Finnish health‐care setting may limit generalizability to systems with different health‐care structures and recording practices.

In conclusion, the results of our study provide novel insights into medical conditions associated with incident EOAD. In particular, brain injuries, alcohol‐related disorders, delirium, thyroid disorders, and epilepsy were significantly more prevalent among EOAD patients, especially during the 1 to 5 years preceding the diagnosis. In contrast, common cardiovascular conditions did not show increased prevalence in EOAD compared to matched controls. These findings suggest that EOAD may be associated with a distinct comorbidity profile, differing from that of LOAD. Further research is warranted to explore the underlying mechanisms and temporal relationships between these comorbidities and EOAD pathogenesis.

## CONFLICT OF INTEREST STATEMENT

The funders of the study had no role in study design, data collection, data analysis, data interpretation, or writing of the report. E. Solje has served on the advisory board of Novartis, Eisai, Eli Lilly, and Roche; served as a consult for Novo Nordisk, EISAI, Eli Lilly, Bioarctic, and Roche; received honoraria for lectures from Lundbeck, BioArctic, Eli Lilly, Novo Nordisk, and Roche; and received travel support from Eli Lilly. J. Krüger has served on the advisory board of Novartis, Nutricia, Bioarctic, Eisai, Eli Lilly, and Roche; received honoraria from lectures from Bioarctic, Eisai, and Eli Lilly; and received support for congress participation from Merck, Eisai, and Eli Lilly. K. Katisko has received honoraria from lectures from Lundbeck. Other authors report no conflicts of interests. Author disclosures are available in the Supporting Information.

## CONSENT STATEMENT

Informed consent was not necessary.

## Supporting information



Supporting Information
